# Nanothorn electrodes for ionic polymer-metal composite artificial muscles

**DOI:** 10.1038/srep06176

**Published:** 2014-08-22

**Authors:** Viljar Palmre, David Pugal, Kwang J. Kim, Kam K. Leang, Kinji Asaka, Alvo Aabloo

**Affiliations:** 1 Department of Chemical and Materials Engineering, University of Nevada, Reno, Nevada, U.S.A; 2 Active Materials and Processing Laboratory, Department of Mechanical Engineering, University of Nevada, Reno, Nevada, U.S.A; 3 Active Materials and Smart Living Laboratory, Department of Mechanical Engineering, University of Nevada, Las Vegas, Nevada, U.S.A; 4 Electroactive Autonomous Systems Laboratory, Department of Mechanical Engineering, University of Utah, Salt Lake City, Utah, U.S.A; 5 Health Research Institute, National Institute of Advanced Industrial Science and Technology, Ikeda, Osaka, Japan; 6 Intelligent Materials and Systems Laboratory, Institute of Technology, University of Tartu, Estonia

## Abstract

Ionic polymer-metal composites (IPMCs) have recently received tremendous interest as soft biomimetic actuators and sensors in various bioengineering and human affinity applications, such as artificial muscles and actuators, aquatic propulsors, robotic end-effectors, and active catheters. Main challenges in developing biomimetic actuators are the attainment of high strain and actuation force at low operating voltage. Here we first report a nanostructured electrode surface design for IPMC comprising platinum nanothorn assemblies with multiple sharp tips. The newly developed actuator with the nanostructured electrodes shows a new way to achieve highly enhanced electromechanical performance over existing flat-surfaced electrodes. We demonstrate that the formation and growth of the nanothorn assemblies at the electrode interface lead to a dramatic improvement (3- to 5-fold increase) in both actuation range and blocking force at low driving voltage (1–3 V). These advances are related to the highly capacitive properties of nanothorn assemblies, increasing significantly the charge transport during the actuation process.

Ionic polymer-metal composites (IPMCs) are unique electroactive polymers (EAPs) that have received enormous research interest over the past decade owing to their soft and flexible structure, relatively large electromechanical bending, and low driving voltage (<4 V)^1^. With the ability to operate in an aqueous environment and mimic closely the motion of biological muscles, IPMC materials are particularly attractive in the field of biomimetics, including underwater robotics, artificial muscles, and biomedical and human affinity applications, such as microsensors and active catheters^2^,^3^,^4^,^5^,^6^,^7^.

Typical IPMC material consists of a thin ionic polymer (ionomer) membrane, such as Nafion®, plated on both sides with noble metal (Pt or Au) layers to serve as electrodes^8^,^9^. The membrane also contains water as a working solvent and mobile cations, typically Li^+^ or Na^+^, that are balanced by fixed anionic groups in the polymer chains. It is understood that applying a voltage across the electrodes causes the migration of hydrated cations toward the negatively charged electrode. The transport of the electrolyte leads to an expansion of the polymer near the cathode and contraction near the anode, resulting in bending actuation of IPMC^9^,^10^,^11^. Also, mechanical deforming of IPMC causes the charge redistribution in the material, allowing it to function as a mechanoelectrical sensor^12^,^13^.

Among the variety of metallic^14^,^15^,^16^,^17^ and non-metallic^18^,^19^,^20^ electrode materials explored for IPMC fabrication, the noble metals such as Pt and Au still remain the ones offering one of the best electrical conductivity and electrochemical stability^21^, making them an excellent choice for small-scale underwater robotic applications where fast actuation responses and corrosion resistance are required. However, while a number of studies have concerned the electric resistance effects of these electrodes^22^,^23^, the effects of electrode surface structure have been largely neglected. As a result, there exists a poor understanding of how to manipulate and control the surface structure of noble metal electrodes, and how the electrode surface profile would affect the IPMC transduction. Our recent finite element simulations^24^ indicate that the shape of the electrode surface profile can have a significant impact on the electromechanical performance of IPMC. The calculations show that by designing the electrode surface as a Koch fractal structure^25^ can considerably increase the total transported charge during the actuation and thereby increase the bending strain of IPMC. However, the conventional platinum electrodes have rather flat surface profile that results in a limited charge accumulation at the electrode interface during the actuation.

In this study, we developed a novel nanostructured electrode surface design for IPMC, composed of platinum nanothorn assemblies. These assemblies, synthesized through electroless plating (also referred to as impregnation-reduction) method, consist of multiple sharp tips and edges radiating outward from the center of assembly. Pt nanoparticles with sharp tips and edges are very rare^26^ and have not been prepared by electroless deposition method or used in EAP application before. Our newly designed IPMC actuators with nanothorn assembly electrodes show dramatically enhanced electromechanical performance over conventional Pt electrodes. A 3- to 5-fold increase in both actuation range and peak blocking force is achieved at 1–3 V driving voltage. These characteristics are related to the unique ability of nanothorn assemblies to efficiently accommodate charges at its interface, allowing higher charge transport during the actuation. The nanothorn assemblies having very fine and sophisticated structure increase significantly the effective surface area of the electrode, enabling more electrolyte ions migrate to its interface boundary. The broader goal of this study is to improve the understanding of the role of electrode surface morphology in the electromechanical coupling of IPMC and provide the basis to advance the performance of IPMC material through surface manipulation of noble metal electrodes. Our newly developed nanostructured Pt electrodes not only advance the performance of IPMC actuators, but can be also beneficial in polymer electrolyte membrane (PEM) fuel cell and various gas sensor applications where electrodes with large specific surface area are desired.

## Results and Discussion

Eight sets of IPMC samples with different Pt electrode surface structures were prepared by varying the number of impregnation-reduction cycles (referred to as primary platings) from 1 to 8 in electroless plating process. A detailed description is provided in the Methods section. The number of subsequent chemical deposition cycles (referred to as secondary platings) was kept constant and one plating was applied to equalize the electrode conductivity of the samples. The notation of the prepared IPMCs and applied platings are listed in Table 1.

### Electrode surface structure

**Figure 1:**
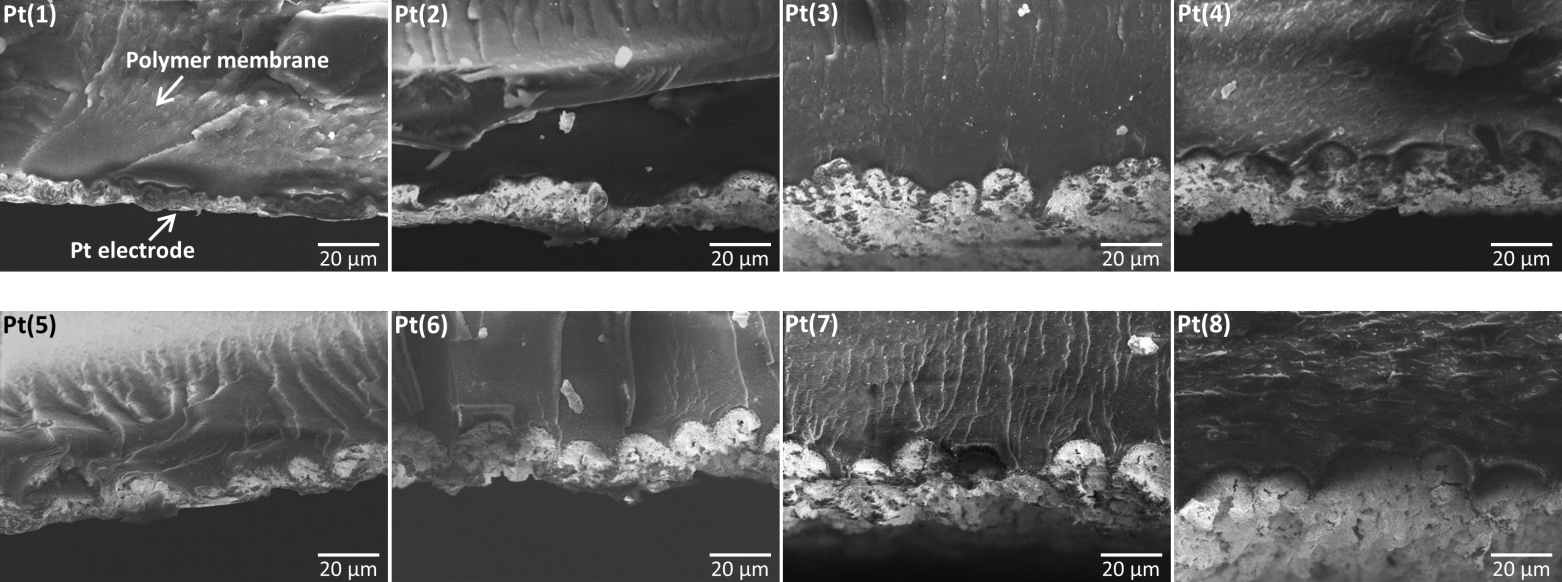
Cross-sectional morphology of IPMCs. SEM cross-sectional micrographs for different IPMC samples (Pt(1)...Pt(8)) at 1k × magnification, showing the deposited Pt electrode layer at the surface of ionomer membrane.

Figure 1 shows the cross-sectional scanning electron microscope (SEM) images of the prepared IPMC samples. The micrographs were taken at 1k × magnification from one side of the cross-section and show the deposited Pt electrode layer at the surface of the polymer membrane in case of each sample. It can be seen that the electrode layer grows in thickness with increasing the number of primary plating cycles from 1 to 8 (samples Pt(1)…Pt(8)). Also, noticeable changes in the electrode surface profile can be observed. The sample Pt(1) has a rather uniform and flat electrode layer, while the samples prepared with higher number of Pt platings exhibit large bumps at the inner surface of the electrode. It should be noted that the uneven and rough regions on the polymer are due to fracturing the samples in liquid nitrogen prior to SEM observations. Also, it is important to note that due to fracturing the cross-section cuts are not perfectly perpendicular to the IPMC surface. Therefore, when the cross-section is aligned perpendicular to the electron beam, the image can also reveal electrode outer surface from a side, making the electrode appear thicker, such as in case of Pt(3).

**Figure 2:**
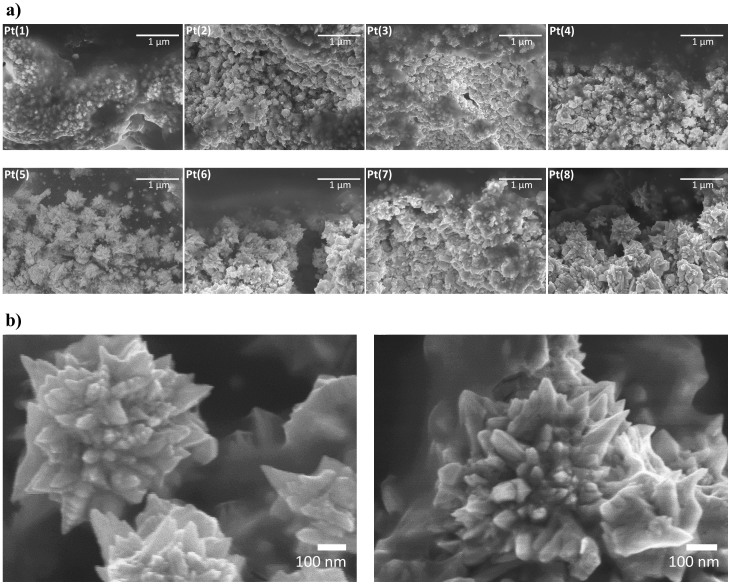
Electrode surface structure. (a) SEM cross-sectional images showing the polymer-electrode interface of IPMCs Pt(1)…Pt(8) at 30k × magnification. (b) Detailed view of nanothorn assemblies at 100k × and 110k × magnification for sample Pt(8).

Figure 2 provides a detailed view of the polymer-electrode interface for different IPMC samples at 30k × magnification. It can be seen that the electrode interface is composed of Pt nanoparticles that gradually become larger with increasing the primary plating cycles. Starting from 5th plating (sample Pt(5)), the platinum particles with multiple sharp tips and edges – called ‘nanothorn assemblies' are formed that become larger and more developed with further increment of plating cycles. The IPMC prepared with eight platings (sample Pt(8)) already shows rather large nanothorn assemblies with well-developed structure at the electrode interface (Fig. 2b). There are very few reports concerning Pt nanoparticles with sharp tips. Tian *et al.* have prepared similar nanostructures on a glassy carbon substrate using electrodeposition method^27^. Our as-synthesized nanothorn assemblies are larger and more developed, and created electrolessly via impregnation-reduction process at the polymer-electrode interface. To the best of our knowledge, such nanostructured Pt assemblies with sharp tips and edges have not been synthesized through electroless plating method or used in EAP application before.

**Figure 3:**
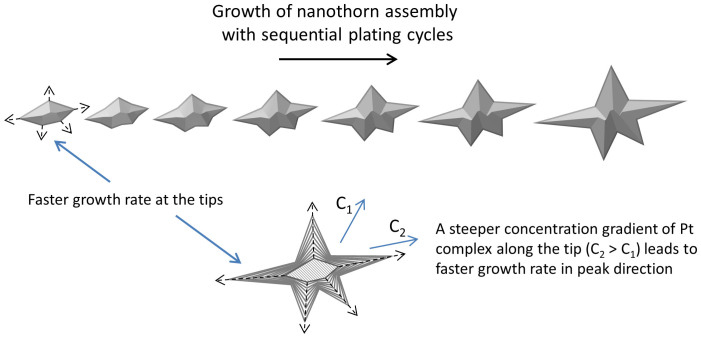
Growth of “nanothorn” assembly. Schematic illustration of the growth of Pt nanothorn assembly with consecutive primary plating cycles. The dashed arrows indicate faster growth rate at the corners of the particle.

The mechanism of formation of Pt nanothorn assemblies is not well understood. However, the dendritic crystal structures generally form due to growth instabilities when the growth rate is limited by the rate of diffusion of atoms to a surface^28^. There also has to be a concentration gradient from a solution to the equilibrium at the interface. Deposited metal particles by nature contain surface defects and imperfections such as bumps and tips. A corner or tip on the deposited particle results in a steeper concentration gradient and thereby increases the diffusion rate, leading to a faster growth of the structure at the tip and eventually formation of peak^28^. The growth of Pt nanothorn assembly with consecutive impregnation-reduction cycles is illustrated in Fig. 3.

**Figure 4:**
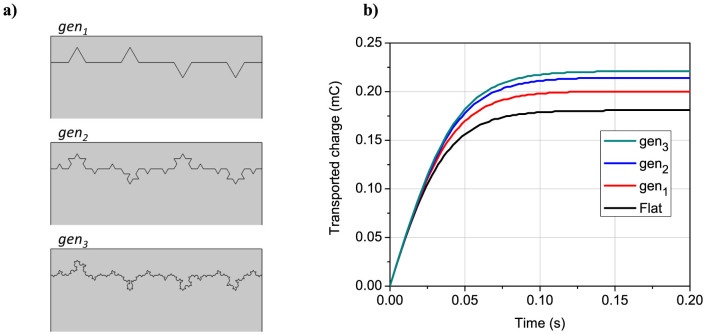
Fractal electrode simulations. (a) Fractal electrode surfaces used in the simulations (domain width = 40 µm), (b) Calculated transported charge in case of flat vs. *gen_1_*…*gen_3_* fractal electrodes at an applied DC voltage of 1 V.

To fundamentally understand the role of electrode surface structure in IPMC transduction, a series of finite element simulations was carried out with different electrode surface profiles using our recently developed physics-based electromechanical model^29^,^30^,^31^. The Koch fractal geometry^25^ was implemented in the model to mathematically describe the polymer-electrode interface in IPMC. The classic Koch fractal algorithm is based on dividing a line segment into three and replacing the middle segment by two sides of equilateral triangle. This procedure is recursively repeated for each of new segments. The fractals were designed random directional, i.e. 50% chance for each triangle to be constructed on top or bottom of the segment. The generation of random directional fractal electrode surfaces (generations noted as *gen_1_*…*gen_3_* based on the fractal depth 1…*n*) is illustrated in Fig. 4a. Due to the complicated geometry of *gen_2_* and *gen_3_* fractals, the domain width for the calculations was chosen very narrow – only 40 µm. Figure 4b shows the simulated transported charge in case of electromechanical transduction of IPMC with an applied voltage of 1 V for flat and fractal electrodes. It can be seen that the transported charge in case of fractal electrodes is considerably higher compared to the flat electrodes and increases with the generation depth of fractals. Due to the complicated geometry of the electrodes and resulting computational cost, the body force and displacement were not considered. However, based on the model^29^,^30^,^31^, higher charge density at the electrodes results in a larger displacement. Thus, the simulations suggest that increasing the electrode interfacial surface area by generating fractals (or dendrites) can improve the electromechanical output of IPMC.

### Electromechanical performance of IPMC actuators

**Figure 5:**
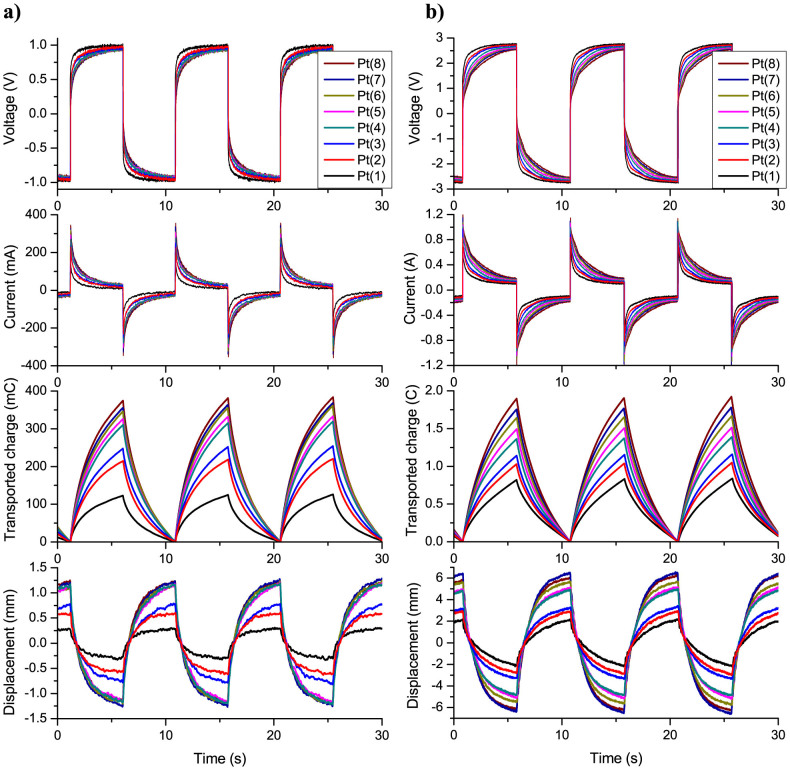
Actuation performance of IPMCs. Measured voltage, current, transported charge and displacement responses for IPMCs with different electrode surface structures (a) at 0.1 Hz, ±1 V and (b) at 0.1 Hz, ±3 V AC square-wave input.

**Figure 6:**
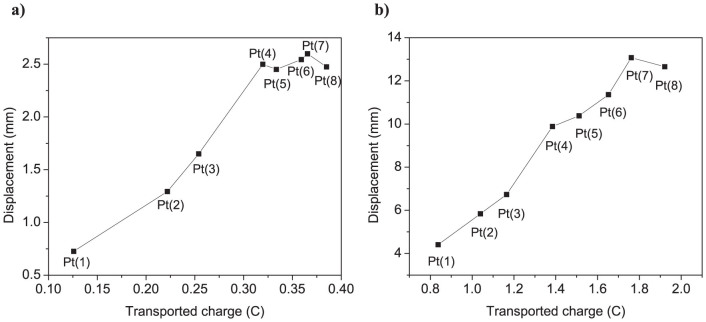
Transported charge/displacement correlation. Peak-to-peak displacement versus transported charge (a) at 0.1 Hz, ±1 V and (b) at 0.1 Hz, ±3 V AC square-wave input.

Figure 5a shows the measured voltage, current, transported charge and corresponding displacement responses in time for IPMCs at ±1 V AC square-wave input at 0.1 Hz. Although the IPMCs of this type are typically operated at input voltages between 2–4 V, a lower voltage (1 V) was used in order to avoid electrochemical processes (i.e. electrolysis of water) – the lost current, to allow more exact analysis of the data in terms of the transported charge. It can be seen that the current as well as the transported charge and displacement are the lowest in case of actuator Pt(1) and increase significantly with the number of plating cycles (or growth of nanothorn assemblies). The peak displacement of actuator Pt(3) is already three-fold compared to that of Pt(1). However, it can be noticed that after the 4th plating the displacement reaches plateau, while there is still a noticeable increase in the transported charge with the number of plating cycles (see Fig. 6a). This is related to low input voltage (1 V) and resulting low actuation force which apparently is too low to overcome the increasing flexural stiffness of the samples with higher number of Pt platings (see Table 1). Overall, the measurements show that the growth of nanothorn assemblies with repeated impregnation-reduction of Pt leads to a higher transported charge and larger displacement of IPMC. These results are in good agreement with our theoretical prediction (see Fig. 4)^24^.

The displacement measurements were also performed at higher voltages in order to see how the transported charge correlates with the actuation performance at normal operating voltages (2–3 V). Figure 5b shows the measured voltage, current, transported charge and displacement responses at ±3 V AC square-wave at 0.1 Hz. As can be seen, the differences in the measured responses in case of different samples are more apparent compared to measurements at ±1 V AC input (Fig. 5a). The peak currents are higher and decay at a slower rate as the number of platings is increased, indicating a higher double-layer charging. It should be noted that the measured current response at a given voltage also includes the charge transfer associated with faradaic processes – electrolysis of water^32^, which does not contribute to the actuation of IPMC. Therefore, exact evaluation of the actuation performance in terms of transported charge is complicated at higher input voltages (>1.8 V). Nonetheless, the measured current/transported charge data correlates well with the displacement response of the samples (Fig. 6b). As compared to the measurements at ±1 V AC (Fig. 6a), the differences in the transported charge and displacement are more pronounced, especially for the IPMCs with higher number of primary platings, i.e. actuators Pt(4)…Pt(8). While there is a steady increment in the charge transport with the number of added platings, the displacement increases up to seventh plating, and the actuator with eight platings (Pt(8)) already shows slightly less displacement than Pt(7). This can be related to the increasing flexural stiffness of the material that starts to limit the actuation performance (see Table. 1). Overall, the data shows that increasing the number of impregnation-reduction cycles from 1 to 7 results in an improvement of displacement amplitude more than 3 times.

**Figure 7:**
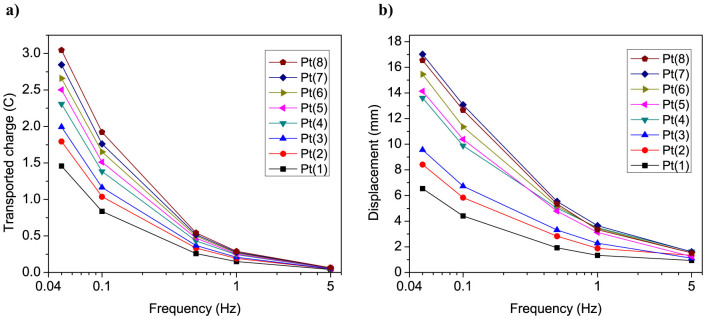
Actuation performance at different frequencies. Frequency dependences of (a) total transported charge and (b) corresponding displacement of IPMCs at ±3 V AC square-wave input between 0.05–5 Hz.

Figure 7 shows the transported charge and corresponding peak-to-peak displacement of Pt-IPMCs as a function of frequency from 0.05 to 5 Hz at ±3 AC square-wave input. The transported charge and the displacement values were calculated by taking an average over minimum of 6 actuation cycles at each measured frequency. As can be seen, the transported charge data correlates well with the actuation performance of IPMCs in the tested frequency range. It can be noticed that at higher frequencies (>1 Hz) the effect of plating cycles on the transported charge and corresponding displacement response is relatively low. This is due to the limited charging time at higher actuation frequency that prevents utilizing the larger interfacial area of the electrodes in case of the samples with higher number of platings. At 0.5 Hz and below, there is a notable increase in the charge transport and displacement response with increasing the number of plating cycles. The actuator response time is suitable for various biomimetic applications, such as for artificial muscle fins and control surfaces used for locomotion and maneuvering of bio-inspired autonomous underwater robotic systems^7^.

**Figure 8:**
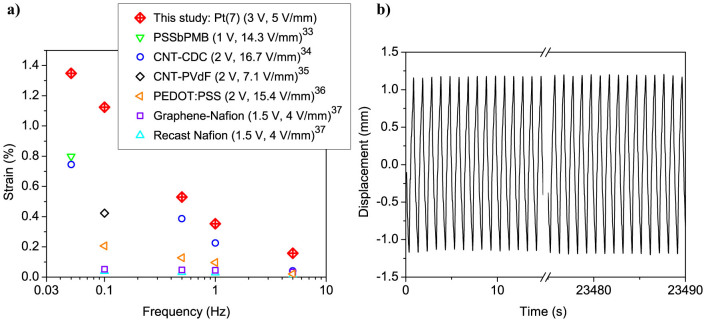
Comparison with other EAPs, and cycle life of the new IPMC actuator. (a) Frequency dependence of the bending strain for IPMC with nanothorn electrodes compared with other ionic EAP actuators reported in the literature. The parameters in the brackets indicate the applied voltage and field strength across the actuator thickness. (b) Cycle life of IPMC with nanothorn electrodes (Pt(7)) under continuous operation at ±2 V AC square-wave input at 1 Hz.

Figure 8a shows the bending strain of the actuator Pt(7) as function of frequency from 0.05–5 Hz at ±3 V AC square-wave input, in comparison with other ionic EAP actuators reported in the literature^33^,^34^,^35^,^36^,^37^. It is important note that the actuators fabricated in this study and elsewhere differ greatly in dimensions, particularly in thickness that ranges from 70 µm to 0.6 mm. Since the displacement output of the EAPs is highly dependent on the intensity of the applied electric field, the different actuators are also compared in terms of the effective field strength (V/mm), taking into account the actuator thickness (across which the voltage is applied). It can be seen that IPMC with nanothorn assembly electrodes operated at relatively low electric field (5 V/mm) exhibits notably higher actuation performance compared with previously reported EAP actuators. In addition to outstanding displacement performance, the new IPMC actuators with nanothorn electrodes also offer a long cycle life. Figure 8b demonstrates the long-term durability of IPMC with nanothorn electrodes under continuous operation in water at ±2 V AC square-wave input at 1 Hz. The actuator shows repeated actuation over 23000 cycles with no decrease in the displacement amplitude.

**Figure 9:**
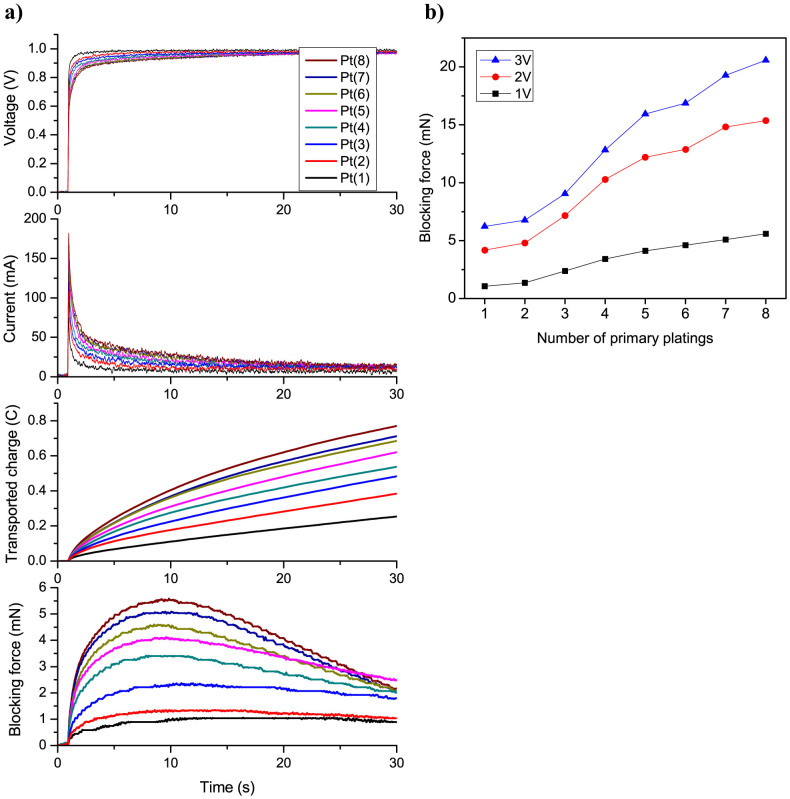
Blocking force performance of IPMCs. (a) Voltage, current, transported charge and blocking force responses in time at 1 V DC input. (b) Peak blocking force vs. the number of plating cycles at different input voltages (1–3 V DC).

The blocking force, which characterizes the generated electromechanical force at IPMC tip at zero displacement, was examined at different driving voltages (1–3 V DC). Figure 9a shows the measured voltage, current, transported charge and corresponding blocking force response in time for different IPMC samples at 1 V DC input. The data shows that increasing the impregnation-reduction cycles leads to a notable increase in the total transported charge and resulting blocking force performance of IPMC. These results are in accordance with the displacement measurements discussed earlier. Figure 9b presents the peak blocking force of IPMCs as a function of primary plating cycles at different input voltages (1–3 V DC). As can be observed, there is a significant increase in the peak blocking force with the growth of nanothorn assemblies, especially at the higher driving voltages (2–3 V). However, the most dramatic increase in the measured force response occurs from the 2nd to 5th plating cycle. The overall improvement in the blocking force performance is more than 5 times in case of 1 V DC and more than 3 times in case of 3 V DC input with growth of nanothorn assemblies.

### Electrochemical properties

**Figure 10:**
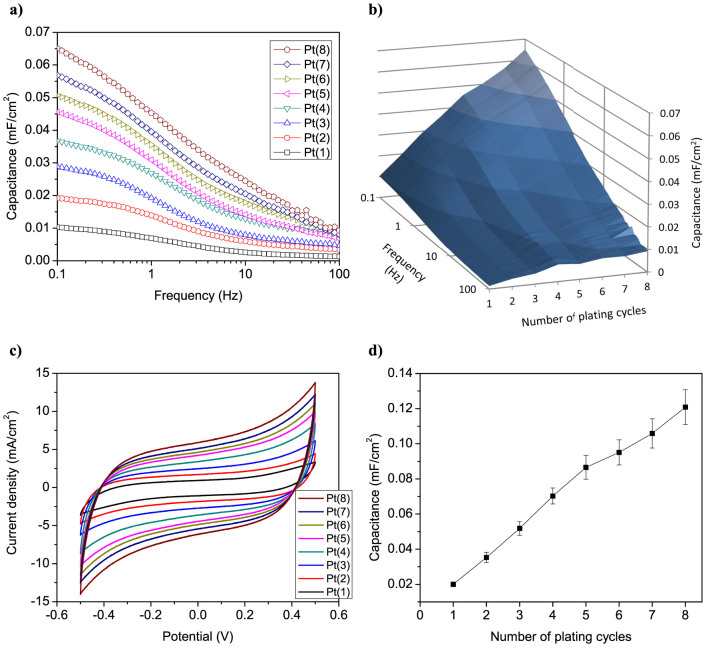
Capacitive properties of Pt nanothorn assemblies. (a) Frequency dependence of differential capacitance for IPMCs with different electrode surface morphologies at 0.1 V DC bias with a 10 mV AC perturbation. (b) An alternative representation of the data: EIS capacitance vs. frequency vs. primary plating cycles. (c) Cyclic voltammograms measured at a potential scan rate of 50 mV/s. (d) Charging-discharging capacitance of IPMCs vs. primary plating cycles.

The cyclic voltammetry (CV) and electrochemical impedance spectroscopy (EIS) techniques were employed to evaluate the interfacial surface of different IPMC electrodes in terms of capacitance. The electric double-layer capacitance describes directly the electrode interfacial area and its effectiveness to accommodate charges at the polymer-electrode boundary. Figure 10a shows the frequency dependence of the differential capacitance for different IPMC samples, obtained from EIS measurements in the frequency range of 0.1–100 Hz with an AC perturbation of 10 mV and DC bias of 0.1 V in two-electrode cell. The measured data shows that increasing the primary plating cycles and thereby developing the nanothorn assemblies at the electrode interface leads to a notable increase in the double-layer capacitance of IPMC at the lower frequencies (*f* < 10 Hz), indicating an enlarged interfacial surface area of nanostructured electrodes. A higher charge accumulation in case of more developed surface geometry is in accordance with the fractal electrode simulations (see Fig. 4).

The charging-discharging capacitance of IPMCs with different electrode surface structures was determined using cyclic voltammetry in a potential range of −0.5 to 0.5 V at the scan rate of 50 mV/s in two-electrode cell. The measured cyclic voltammograms in Fig. 10c indicate non-faradaic capacitive current behavior. IPMC in principle is similar to electrochemical capacitor that stores energy in electric double layer using high surface area electrodes and electrolyte. It can be seen in Fig. 10c that increasing the number of plating cycles leads to a considerably higher current densities during the charging and discharging, which can be expected as more charges are involved in the double-layer formation due to the larger interfacial surface area of electrodes. The charging-discharging capacitance values were determined from the measured cyclic voltammograms according to the equation (3) and are plotted against the number of plating cycles, as shown in Fig. 10d. As can be seen, there is a steady and almost linear increase in the capacitance with the increment of plating cycles (growth of nanothorn assemblies). The improvement in the capacitance is six times (0.02 mF/cm^2^ to 0.121 mF/cm^2^) with increasing the plating cycles from 1 to 8. These results are consistent with EIS measurements as well as with the SEM data, and are in good agreement with electromechanical performance data of IPMCs. The capacitance measurements demonstrate that careful control of the synthesis parameters of electroless plating process allows good control over the interfacial area of Pt nanothorn assembly electrodes.

## Methods

### Chemicals and materials used

Perfluorinated sulfonic acid ionomer film (thickness 0.6 mm) was purchased from GEFC Co., Ltd and was used as a membrane material for IPMC fabrication. The GEFC membrane has a similar chemical structure and properties to Nafion® and has shown one of the best performances among the ion-exchange membranes tested for IPMC application^38^. The following reagents were of analytical grade and were used as received: tetraammineplatinum(II) chloride monohydrate (Pt(NH_3_)_4_Cl_2_·H_2_O, 98%, Aldrich), sodium borohydride (NaBH_4_, 98%, Sigma), hydroxylamine hydrochloride (H_2_NOHHCl, 98%, Sigma), hydrazine monohydrate (H_2_NNH_2_·H_2_O, 98%, Sigma), and lithium chloride (LiCl, 99%, Sigma).

### Conditioning of the membrane

Ionomer membrane was pretreated by roughening both surfaces with emery paper (800 and 1000 Grit) to enhance the physical bonding between the polymer and metal electrode layer. Thereafter, the ionomer membrane was cleaned in 3 wt% hydrogen peroxide (H_2_O_2_) solution at 70°C for 45 min and subsequently in 1 M sulfuric acid (H_2_SO_4_) solution at the same temperature and duration. Finally, the membrane was cleaned in deionized water at 70°C for 30 min to remove acid residues.

### Fabrication of IPMC actuators

At first, platinum particles were deposited at the inner surface of the membrane using an impregnation-reduction method^9^,^39^. The ionic polymer was soaked in 15–25 mM platinum salt (Pt(NH_3_)_4_Cl_2_·H_2_O) solution for 3–4 h to impregnate the ionomer with Pt^2+^ complex ions. After rinsing with deionized water, the membrane was immersed in 350 ml of aqueous solution at 60°C, containing 0.2 g of sodium borohydride (NaBH_4_) as a reducing agent and 0.3 ml of ammonium hydroxide (NH_4_OH) for pH adjustment. While under moderate stirring, 0.2 g of NaBH_4_ was added in every 30 min for 2 h. During this process, the platinum salt in the polymer is chemically reduced to metallic form (Pt^0^) at the inner surface of the membrane. After that, the obtained composite was cleaned in 1 M H_2_SO_4_ solution for 30 min at 70°C and then in deionized water at the same temperature and duration. The mentioned impregnation-reduction cycle was repeated multiple times to increase the electrode thickness and promote the growth of nanothorn assemblies.

As for next step, the platinum particles were deposited on the outer surface of the composite using a chemical deposition method (referred to as secondary plating). This step is implemented to further increase the electrode surface conductivity. The membrane was immersed in platinum salt (Pt(NH_3_)_4_Cl_2_·H_2_O) solution and while moderately stirring, mild reducing agents, hydrazine monohydrate (H_2_NNH_2_·H_2_O) and hydroxylamine hydrochloride (H_2_NOHHCl), were added in every 30 min for 4 h to deposit the Pt particles on the surface of the membrane. After typical cleaning procedure in H_2_SO_4_ and deionized water, the chemical deposition step was repeated, depending on electrode conductivity. The reason why the sequential impregnation-reduction process described earlier leads to deposition of Pt at the inner membrane surface while the secondary plating step deposits Pt particles at the outer surface, relies in the fundamental difference in these processes. In the first case, the Pt complex that is contained in the membrane diffuses towards the membrane surface during reduction step (driven by concentration gradient) where it reacts with the reducing agent (NaBH_4_) from a surrounding solution. In case of secondary plating process, already Pt coated IPMC (not containing Pt complex) is immersed in a Pt complex solution with reducing agents. Therefore, the Pt that is reduced from the solution deposits predominantly on top of the IPMC surface^9^,^39^.

Using the aforementioned procedures, eight sets of IPMC samples were prepared by varying the number of impregnation-reduction cycles (referred to as primary platings) from 1 to 8. In all cases the time intervals during the impregnation-reduction process were precisely 3.5 h for impregnation in Pt complex, 2 h for reduction and 2 h for cleaning process for each plating cycle. The number of subsequent chemical deposition cycles (secondary platings) was kept constant and one plating was applied to equalize the electrode surface conductivity of the samples. The prepared samples were cut into 50 mm × 10 mm rectangular shape and ion-exchanged to Li^+^-form by soaking in lithium chloride (1 M LiCl) solution for overnight.

### Electromechanical characterization

**Figure 11:**
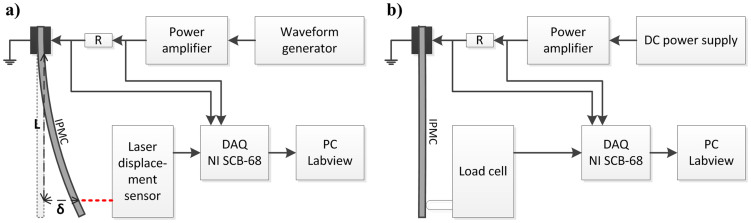
Experimental setups. Schematic of the setup used for (a) displacement and (b) blocking force measurement of IPMC.

The electromechanical responses such as displacement and blocking force of the prepared IPMCs were measured by a laser displacement sensor (optoNCDT-1401, Micro-Epsilon) and a load cell (GSO-30, Transducer Techniques) with a sample size of 50 mm × 10 mm in the test setup composed of a signal generator (FG-7002C, EZ digital), a power amplifier (LVC-608, AE Techron), a DC power supply (CPS250, Tektronix), and a DAQ (SCB-68, National Instruments), as illustrated in Fig. 11. IPMC was clamped in a cantilever configuration in water with a free-length of 40 mm. The displacement (*δ*) was monitored at a position of 35 mm away (*L*) from the clamp contacts. The voltage, current and displacement (or blocking force) responses were recorded using National Instruments/LabView 8 data acquisition system. The performances of different IPMCs were analyzed in the terms of the total transported charge (*Q*), calculated from the measured current by integration: 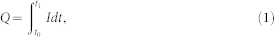
where *I* is the measured current transferred between the times *t_0_* and *t_1_*. The measured displacement (*δ*) was converted to bending strain (*ε*) according to the following equation^9^: 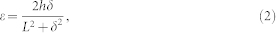
where *h* is the thickness and *L* is the free length of IPMC.

### Electrochemical characterization

Cyclic voltammetry (CV) measurements were conducted using a Radiometer Analytical PGZ-402 potentiostat in a two-electrode cell configuration in a potential range of -0.5 to 0.5 V at the scan rate of 50 mV/s. The double-layer capacitance (*C*) was determined from the cyclic voltammograms using the following equation^40^: 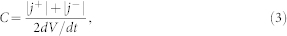
where *j^+^* and *j^−^* are the polarization current densities at 0 V, and *dV/dt* is the potential scan rate.

EIS (differential capacitance) measurements were performed in a frequency range of 0.1 Hz to 100 kHz with an AC perturbation of 10 mV using a Radiometer Analytical PGZ-402 potentiostat. The differential double-layer capacitance for IPMC was determined from EIS measurements according to the following equation^41^,^42^: 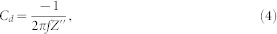
where *f* is the frequency of applied electric potential signal and *Z″* is the imaginary part of the impedance. IPMC is considered as a circuit consisting of a capacitor and resistor in series^43^,^44^.

### Surface characterization and mechanical properties

SEM images were obtained using a Hitachi S-4200 microscope in a secondary electron image mode with accelerating voltages of 5–15 kV. The samples were dried and fractured in liquid nitrogen before SEM observations to obtain clean cut cross-sections. The flexural (bending) modulus of the samples was measured in a three-point bending mode using an Instron universal testing machine (model 5565).

## Author Contributions

K.J.K. conceived the idea and designed the project. V.P. planned and carried out the experiments, analyzed the data and wrote the paper. D.P. and A.A. formulated and performed simulations. K.K.L. helped with experimental work. K.A. helped conceived the idea of making fractal electrodes. All authors discussed the results and commented on the manuscript.

## Figures and Tables

**Table 1:** Notation of IPMC actuators prepared in the study

Notation	No. of primary platings	No. of secondary platings	Measured flexural modulus (MPa)
Pt(1)	1	1	126
Pt(2)	2	1	137
Pt(3)	3	1	139
Pt(4)	4	1	153
Pt(5)	5	1	159
Pt(6)	6	1	174
Pt(7)	7	1	177
Pt(8)	8	1	189
